# Correlation of hemoglobin levels with diabetic retinopathy in US adults aged ≥40 years: the NHANES 2005–2008

**DOI:** 10.3389/fendo.2023.1195647

**Published:** 2023-08-02

**Authors:** Xiao Li, Meirong Chen

**Affiliations:** ^1^ First Clinical Medical College, Shandong University of Traditional Chinese Medicine, Jinan, China; ^2^ Ophthalmology Department, Shandong Hospital of Traditional Chinese Medicine, Jinan, China

**Keywords:** diabetes, diabetic retinopathy, NHANES, hemoglobin, cross-sectional study

## Abstract

**Purpose:**

The aim of this study was to explore the connection between hemoglobin levels and diabetic retinopathy (DR).

**Methods:**

Cross-sectional research used data from the National Health and Nutrition Examination Survey (NHANES) 2005–2008. A multiple logistic regression analysis was performed to investigate the association between DR and hemoglobin levels. Additionally, generalized additivity models and smoothed curve fitting were carried out.

**Results:**

After adjusting for several covariates, there was a negative association between hemoglobin levels and DR in the study, which included 837 participants. The negative association between hemoglobin levels and DR was present in men and women, the obese (BMI > 30), and 60- to 69-year-olds in subgroup analyses stratified by sex, BMI, and age. The association between hemoglobin levels and DR in the normal weight group (BMI < 25) displayed an inverted U-shaped curve with an inflection point of 13.7 (g/dL).

**Conclusion:**

In conclusion, our research reveals that high hemoglobin levels are related to a decreased risk of DR. Ascertaining the hemoglobin levels ought to be regarded as an integral facet of the monitoring regimen for patients with diabetic complications and that the risk of DR is reduced through the detection and management of hemoglobin levels.

## Background

As one of the most significant public health issues in the world, diabetes mellitus (DM) can cause difficulties in numerous organ systems (1). One of the major causes of blindness globally, diabetic retinopathy (DR) is a frequent diabetic consequence that manifests as a series of lesions in the retina brought on by microvascular damage from diabetes (2). The frequency of DR is approximately 33% in Western nations (3, 4). As society continues to evolve, unhealthy human lifestyles may also further increase the prevalence of DR (5). Hemoglobin levels below 13 g/dL in men and 12 g/dL in women are termed anemic (6). In diabetic individuals, anemia may increase the incidence of retinal microangiopathy (7, 8). Although the exact cause of DR is uncertain, early research has indicated that hyperglycemia, hyperlipidemia, and hypertension are all strongly linked to the onset of DR (9). Other research has indicated that anemia (10–12) and hemoglobin levels (13–15) are also linked to DR. However, because of the small number of participants in previous research, as well as the study population’s unique hospital and race background, the link between hemoglobin levels and DR has yet to be completely explored. The NHANES 2005–2008 database was used in this study to further investigate the relationship.

## Materials and methods

### Study population

**Figure 1:**
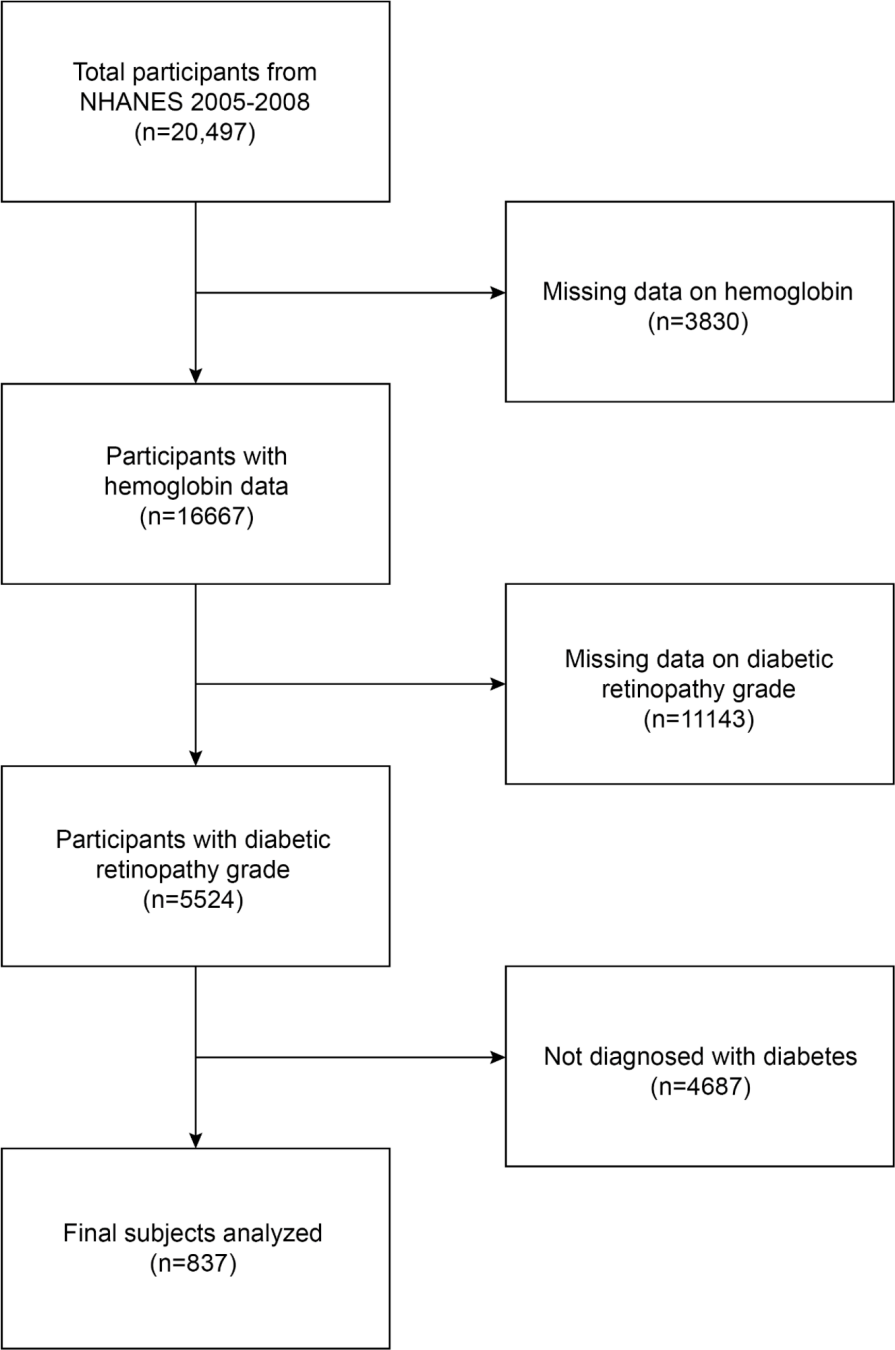
Flowchart of the participant selection. NHANES, National Health and Nutrition Examination Survey.

The National Health and Nutrition Examination Survey (NHANES) database uses a multi-stage stratified sample to collect detailed information on a nationally representative study population, intending to provide objective and realistic statistics based on the health condition of the human body and address emerging health issues in society. In our current study, the 2005–2008 NHANES data survey included 20,497 participants. A total of 3,830 people with missing hemoglobin data, 11,143 people with missing DR grade, and 4,687 people without a diabetes diagnosis were eliminated. In the end, 837 people took part in the investigation (**Figure 1**).

### Ascertainment of diabetic retinopathy

A Canon Non-Mydriatic Retinal Camera CR6-45NM was used to take forty-five-degree non-mydriatic digital photographs of the retina from survey participants 40 years of age and older. People who were blind (unable to see with both eyes open), had eye infections, or wore eye patches in both eyes were disqualified from the retinal imaging component. At least two graders from the University of Wisconsin further rated each photograph. After systematic grading, the eye was graded by a third grader if the initial two graders could not agree on the pathology. A final judgment on the photograph was made by an adjudicator if two out of the three graders could not agree. The NHANES Digital Grading Protocol divided DR severity levels into no retinopathy, mild non-proliferative retinopathy (NPR), moderate-severe NPR, and proliferative retinopathy (PDR). We utilized the status of the eye with the more severe retinopathy in our analysis when retinal pictures for both eyes were available.

### Ascertainment of hemoglobin level

A complete blood count on blood specimens is produced by the Beckman Coulter MAXM equipment in the Mobile Examination Centers (MECs), and it also offers a distribution of blood cells for each participant. An extensive explanation of the laboratory techniques used may be found in the Laboratory Method Files section (https://www.cdc.gov/nchs/nhanes/).

### Covariates

Demographic variables for this study included age, race, gender, body mass index, poverty income ratio, and educational attainment. Anthropometric and laboratory covariates involved in the study included waist circumference, glycated hemoglobin, triglycerides, total cholesterol, HDL cholesterol, and LDL cholesterol. Self-reported daily behaviors and health status were also considered, including smoking at least 100 cigarettes at the time of data collection, drinking at least 12 drinks in any given year, insulin treatment, and duration of diabetes. To determine the duration of diabetes, the person’s age at the time they were first informed they had the disease was deducted from their claimed age at the screening. Responses to the following query, “Were you told on two or more different visits that you had hypertension, also called high blood pressure? “ were used to define hypertension. Those who responded “yes” to the question “Do you drink at least 12 alcohol drinks in any one year?” were defined as a drinker. On the NHANES website (https://www.cdc.gov/nchs/nhanes/), you may find the calculation procedures for each of the aforementioned variables.

### Statistical analyses

All statistical analyses were conducted using R (http://www.r-project.org) and EmpowerStats (http://www.empowerstats.com), with the level of statistical significance set at *p* < 0.05. Categorical variables were described as proportions, whereas continuous variables were described as means with standard deviations (SDs). Differences between participants were evaluated using the chi-squared test (for categorical variables) based on the tertiles of their hemoglobin levels. In subgroups by sex (women/men), age (40–49/50–59/60–69/70 years), and BMI (normal weight/overweight/obesity), the relationship between hemoglobin levels and DR was examined. Model 1 had no variables modified. Age, gender, and race were the key demographic variables that were adjusted in model 2, while all covariates that were used in the study were adjusted in model 3. A weighted generalized additive model and smooth curve fitting were applied to investigate non-linearity.

## Results

### Baseline characteristics

According to the inclusion and exclusion criteria, a total of 837 adults aged ≥40 years were included in this research. The average age was 63.25 ± 10.52 years; 50.2% of these individuals were men, while 49.8% were women. In addition to 19.4% Mexican Americans, there were also 39.9% non-Hispanic white people, 29.6% non-Hispanic black people, 8.4% other Hispanic people, and 2.7% people of other races. The percentage of participants with mild NPR was 64.9%, those with moderate-to-severe NPR was 25.8%, and those with PDR was 9.3%. Based on DR status, the baseline clinical characteristics of the research are described in **Table 1**. In contrast to the non-DR group, the DR group was more likely to be male participants, had a longer duration of DM, a higher proportion of insulin therapy, and higher hemoglobin and glycated hemoglobin levels.

**Table 1:** Characteristics of participants grouped with or without diabetic retinopathy.

	Non-DR	DR	*p*-value
	*N* = 535	*N* = 302	
Age, years	62.90 ± 10.55	63.88 ± 10.45	0.193
Sex, %			0.036
Male	47.10	54.64	NA
Female	52.90	45.36	NA
Race, %			0.069
Non-Hispanic White	42.24	35.76	NA
Non-Hispanic Black	26.36	35.43	NA
Mexican American	19.44	19.21	NA
Other Hispanic	8.79	7.62	NA
Other	3.18	1.99	NA
Education, %			0.257
Less than high school	19.07	23.84	NA
High school	45.42	43.38	NA
More than high school	35.51	32.78	NA
Poverty income ratio	2.36 ± 1.48	2.38 ± 1.40	0.848
Smoked at least 100 cigarettes in life, %			0.673
Yes	53.83	52.32	NA
No	46.17	47.68	NA
Drinker, %	56.07	54.97	0.757
Hypertension, %			0.770
Yes	60.56	61.59	NA
No	39.44	38.41	NA
Duration of diabetes, years	8.35 ± 9.98	15.62 ± 11.26	<0.001
Insulin therapy, %	13.83	42.72	<0.001
Body mass index, kg/m^2^	32.39 ± 7.10	31.55 ± 6.64	0.091
Waist circumference, cm	109.07 ± 14.79	107.67 ± 14.49	0.187
Total cholesterol (mmol/L, mean ± SD)	4.82 ± 1.21	4.83 ± 1.21	0.910
Triglyceride (mmol/L, mean ± SD)	1.96 ± 1.06	1.96 ± 1.09	0.980
HDL (mmol/L, mean ± SD)	1.26 ± 0.36	1.28 ± 0.37	0.378
LDL (mmol/L, mean ± SD)	2.60 ± 0.65	2.61 ± 0.67	0.791
HbA1C, %	6.97 ± 1.53	7.95 ± 1.76	<0.001
Hemoglobin (g/dL, mean ± SD)	13.85 ± 1.51	13.62 ± 1.70	0.047
Retinopathy severity, %			
Mild NPR	NA	64.9	NA
Moderate-to-severe NPR	NA	25.8	NA
Proliferative retinopathy	NA	9.3	NA

Mean ± SD for continuous variables: the *P*-value was calculated by the linear regression model.

(%) for categorical variables: the *P*-value was calculated by the chi-square test.

HbA1c, glycated hemoglobin; NPR, non-proliferative retinopathy; HDL, high-density lipoprotein; LDL, low-density lipoprotein; DR, diabetic retinopathy.

### Relationship between hemoglobin levels and diabetic retinopathy

**Figure 2:**
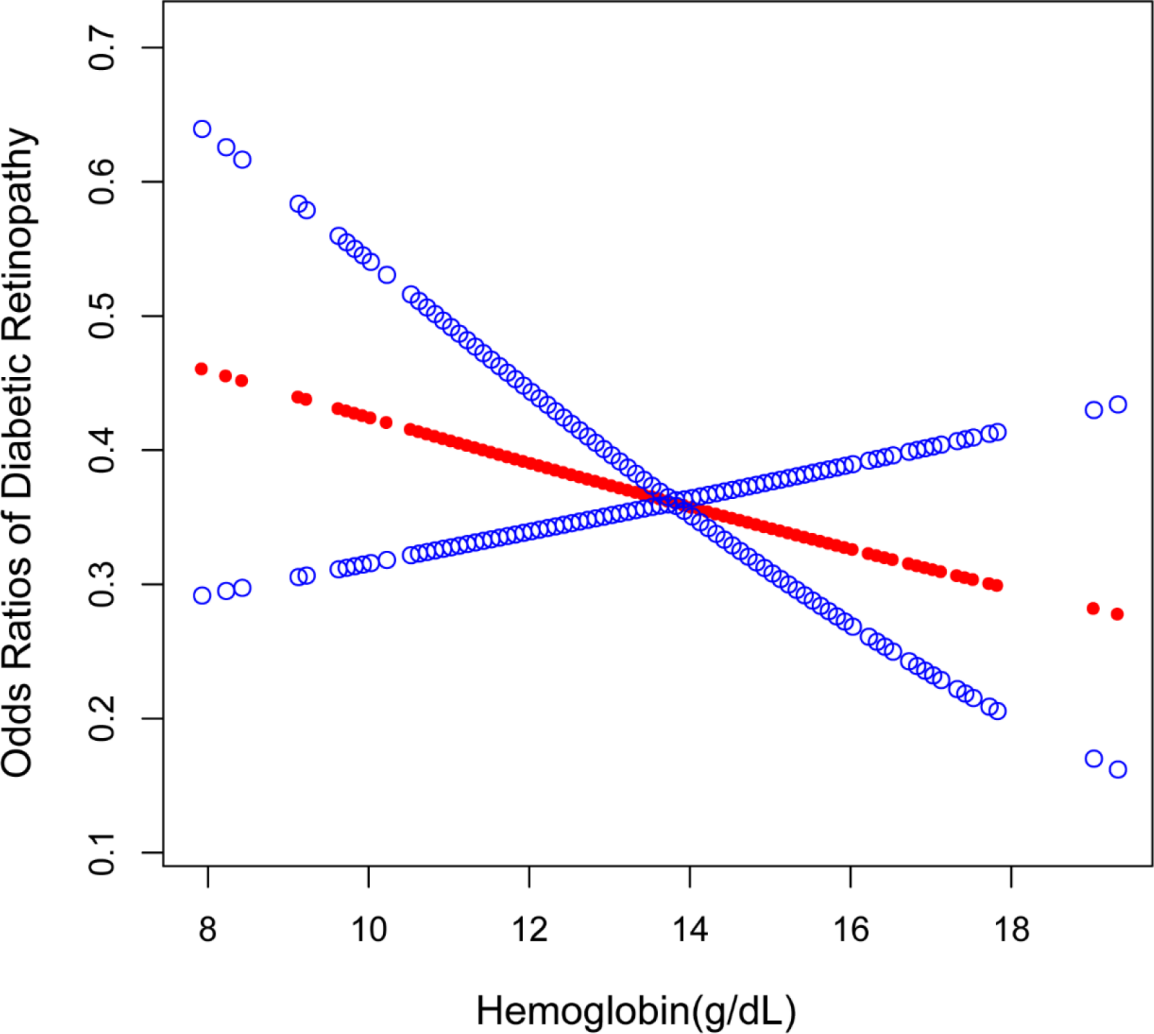
The association between hemoglobin levels and DR. The solid red line represents the smooth curve fit between variables. Blue bands represent the 95% confidence interval from the fit. DR, diabetic retinopathy.

The results of the multiple logistic regression analysis are displayed in **Table 2**. Hemoglobin levels were negatively correlated with DR in the unadjusted model [OR = 0.91, 95% CI: (0.83,1.00), *p* = 0.0472]. Hemoglobin levels continue to have a strong negative relationship with DR in model 2 [OR = 0.89, 95% CI: (0.80,0.98), *p* = 0.0238], after adjusting for gender, age, and race. However, after adjusting for all covariates, this significant association ceased to be significant in model 3 [OR = 0.93, 95% CI: (0.82,1.05), *p* = 0.2608]. The incidence of DR decreased by 36% in model 1 [OR = 0.64, 95% CI: (0.45,0.91), *p* = 0.0124] when comparing the greatest tertile of hemoglobin levels (tertile 3) to the lowest tertile (tertile 1). The negative non-linear relationship between hemoglobin levels and DR is further verified in **Figure 2**.

**Table 2:** Relationship between hemoglobin levels and diabetic retinopathy.

	Model 1 OR (95% CI) *p*-value	Model 2 OR (95% CI) *p*-value	Model 3 OR (95% CI) *p*-value
Hemoglobin (g/dL)	0.91 (0.83, 1.00) 0.0472	0.89 (0.80, 0.98) 0.0238	0.93 (0.82, 1.05) 0.2608
T1	Reference	Reference	Reference
T2	0.78 (0.55, 1.09) 0.1467	0.74 (0.52, 1.06) 0.1059	0.91 (0.60, 1.37) 0.6474
T3	0.64 (0.45, 0.91) 0.0124	0.54 (0.36, 0.82) 0.0041	0.70 (0.43, 1.13) 0.1413
*p* for trend	0.86 (0.76, 0.97) 0.0122	0.82 (0.71, 0.94) 0.0041	0.89 (0.76, 1.04) 0.1514
*Stratified by gender*			
Men	0.86 (0.75, 0.98) 0.0274	0.88 (0.77, 1.02) 0.0872	0.88 (0.75, 1.04) 0.1366
Women	0.84 (0.72, 0.98) 0.0238	0.88 (0.75, 1.03) 0.1104	0.95 (0.79, 1.15) 0.6125
*Stratified by age*			
40–49 years	0.96 (0.76, 1.22) 0.7362	0.90 (0.66, 1.23) 0.5246	0.68 (0.37, 1.26) 0.2211
50–59 years	1.07 (0.88, 1.29) 0.4914	1.03 (0.83, 1.28) 0.7609	0.97 (0.72, 1.31) 0.8455
60–69 years	0.83 (0.71, 0.97) 0.0206	0.78 (0.65, 0.94) 0.0094	0.94 (0.75, 1.17) 0.5678
≥70 years	0.89 (0.75, 1.06) 0.1802	0.88 (0.72, 1.08) 0.2121	0.89 (0.71, 1.13) 0.3340
*Stratified by BMI*			
Normal weight	1.04 (0.80, 1.35) 0.7852	0.91 (0.65, 1.29) 0.5969	0.77 (0.47, 1.26) 0.3050
Overweight	1.00 (0.84, 1.17) 0.9553	1.01 (0.82, 1.24) 0.9490	1.02 (0.80, 1.30) 0.8504
Obese	0.83 (0.74, 0.94) 0.0029	0.82 (0.71, 0.94) 0.0045	0.90 (0.76, 1.06) 0.1951

Model 1: no covariates were adjusted. Model 2: age, gender, and race were adjusted. Model 3: age, gender, race, educational level, BMI, poverty income ratio, waist circumference, alcohol consumption status, duration of diabetes, alcohol consumption status, hypertension status, insulin therapy, HDL, LDL, HbA1C, triglyceride, and total cholesterol were adjusted.

In the subgroup analysis stratified by gender and race, the model is not adjusted for sex and race, respectively.

### Subgroup analysis

**Figure 3:**
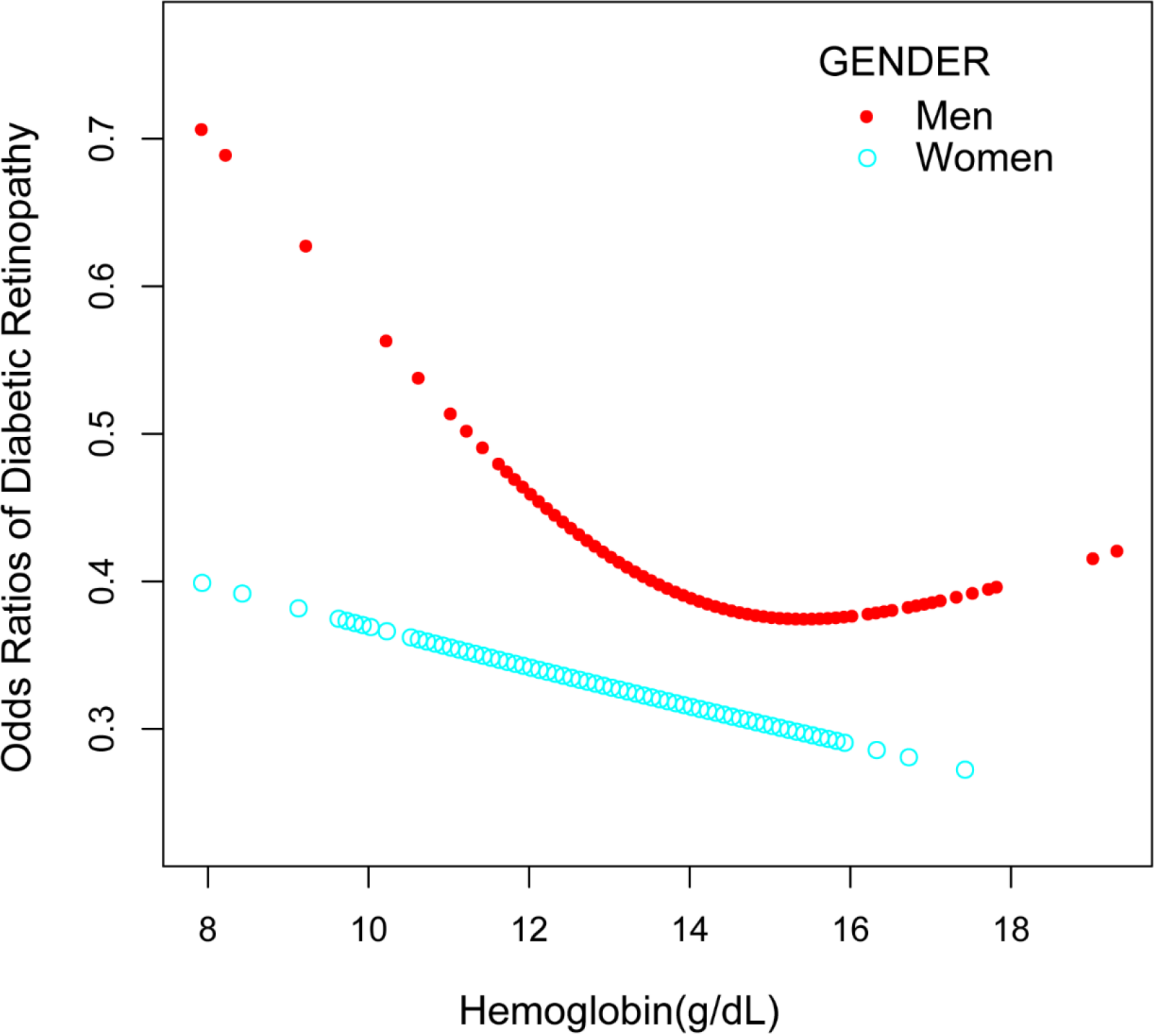
Relationship between hemoglobin levels and DR stratified by gender. DR, diabetic retinopathy.

**Figure 4:**
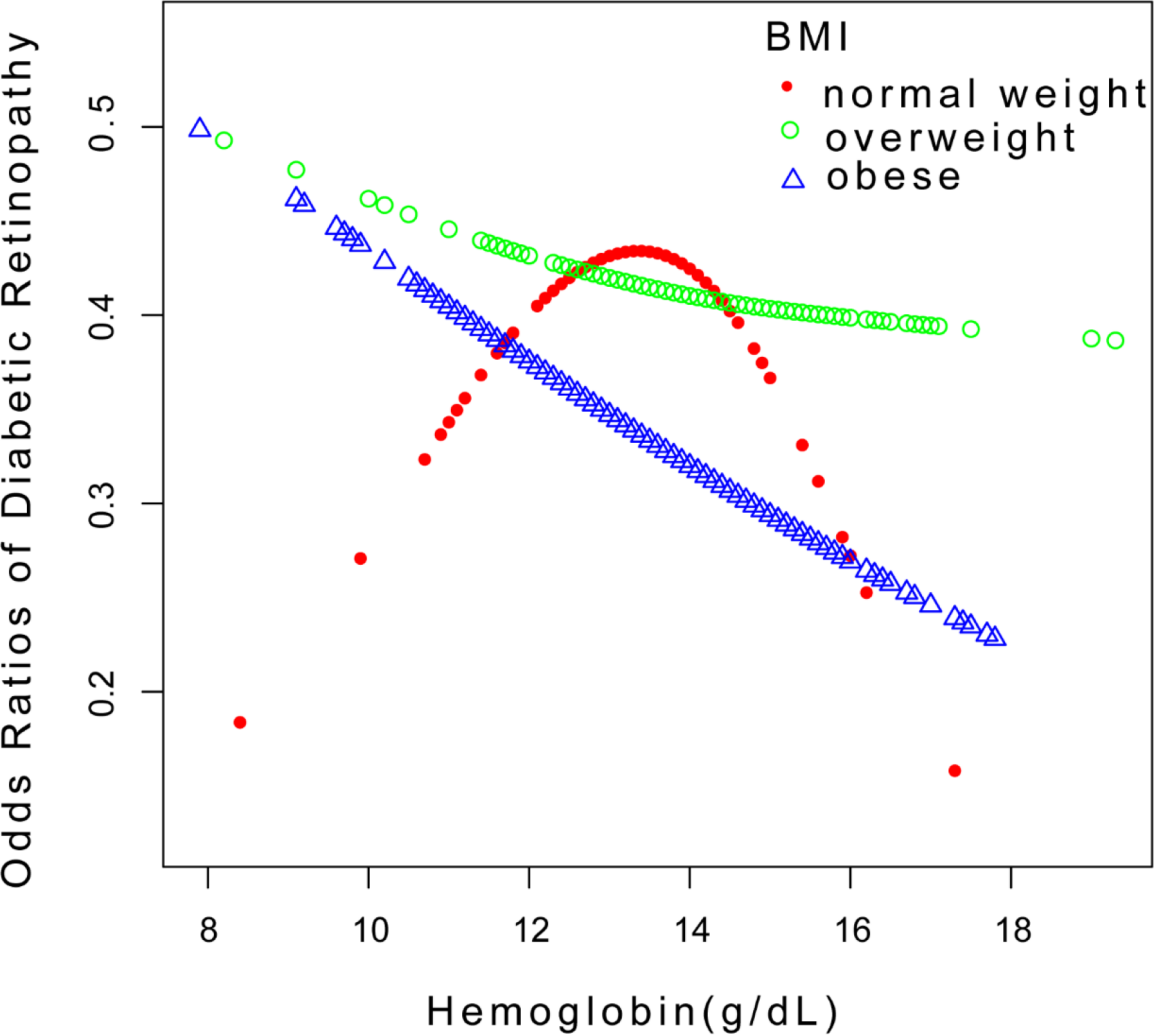
Relationship between hemoglobin levels and DR stratified by BMI. DR, diabetic retinopathy.

Our findings reveal a significant negative association between hemoglobin levels and DR in the unadjusted model for men [OR = 0.86, 95% CI: (0.75,0.98), *p* = 0.0274] in subgroup analyses stratified by gender (**Table 2** and **Figure 3**). Women were similar [OR = 0.84, 95% CI: (0.72,0.98), *p* = 0.0238]. Hemoglobin levels and DR exhibited a significant negative association in the 60–69 age range in both model 1 and model 2 in subgroup analyses stratified by age (**Table 2**). The hemoglobin levels and DR demonstrated a negative significant association in the obese group in both model 1 and model 2 when we conducted a subgroup analysis stratified by BMI (**Table 2**). Additionally, smooth curve fits and generalized additive models were employed to represent the non-linear connection. A two-piecewise linear regression model was used and an inverted U-shaped association was found between hemoglobin levels and DR, with the inflection point in the normal weight group being 13.7 (g/dL) (**Figure 4**). In a non-linear association by gender grouping, men showed a U-shaped curve with inflection points of 12.6 (g/dL) (**Figure 3**), but the log-likelihood ratio was not significant (**Table 3**).

**Table 3:** Threshold effect analysis of hemoglobin levels on diabetic retinopathy using the two-piecewise linear regression model.

Odds ratios of diabetic retinopathy	Adjusted OR (95% CI) *p-*value
Normal weight	
Inflection point	13.7
Hemoglobin < 13.7 (g/dL)	1.24 (0.60, 2.54) 0.5657
Hemoglobin > 13.7 (g/dL)	0.29 (0.09, 0.97) 0.0447
Log likelihood ratio	0.043
Men	
Inflection point	12.6
Hemoglobin < 12.6 (g/dL)	0.41 (0.16, 1.04) 0.0597
Hemoglobin > 12.6 (g/dL)	0.99 (0.80, 1.21) 0.8916
Log likelihood ratio	0.058

Age, gender, race, educational level, BMI, poverty income ratio, waist circumference, alcohol consumption status, duration of diabetes, alcohol consumption status, hypertension status, insulin therapy, HDL, LDL, HbA1C, triglyceride, and total cholesterol were adjusted.

## Discussion

In this study, which included 837 participants who met the nadir criteria, we found a negative linear association between hemoglobin levels and DR. Additionally, we discovered an inverted U-shaped association between hemoglobin levels and DR in a population with normal weight after conducting a subgroup analysis.

The negative association presented a similar trend to a previous study. In a cross-sectional case–control study including 312 people, Traveset et al. discovered that lower hemoglobin levels were related to the occurrence of severe DR and retinal ischemia (13). A prospective investigation conducted in India, involving 306 patients with type 2 diabetes who met the nadir criteria at a specialized hospital, demonstrated a robust association between reduced hemoglobin levels and DR (15). Similarly, utilizing a multiple logistic regression model, Lee et al. discovered a significant inverse relationship between hemoglobin levels and DR risk in a cross-sectional analysis of the Korean population based on the KNHANES database. Specifically, compared to the lowest quartile of hemoglobin concentrations, individuals in the highest quartile exhibited a 62% decrease in DR risk [OR = 0.38; 95% CI: (0.26–0.58)] (16). In our investigation, we found that hemoglobin levels negatively correlated with DR in both model 1 and model 2.

Compared to previous studies, our study further investigated the relationship between hemoglobin levels and DR by conducting subgroup analyses by gender, age, and BMI. That is, our findings not only support the previous conclusion that lower hemoglobin levels are associated with a higher risk of DR but also reveal the association between hemoglobin levels and DR when subgroups are analyzed by BMI. In particular, we discovered a non-linear association between hemoglobin levels and DR in the normal-weight population in the subgroup analysis. We further conclude that the inflection point for hemoglobin is 13.7 (g/dL), meaning that the correlation between hemoglobin and DR is meaningful when hemoglobin levels in normal-weight people reach this threshold. We found this inflection point to be similar to the hemoglobin value that defines anemia (6). This non-linear relationship may be due to the lack of protective mechanisms against the onset of DR in normal-weight people compared to people who are overweight and obese, and so the effect of anemia on DR cannot be mitigated. Specifically, individuals with a greater BMI exhibit elevated levels of C-peptide, which leads to augmented blood flow and wider capillary dispersion (17). A study performed on rats supplemented with C-peptide highlighted noteworthy enhancements in nerve conduction velocity and microvascular blood flow (18). Additionally, C-peptide amplifies the expression of endothelial NO-synthase (eNOS), thereby promoting the secretion of nitric oxide (NO) from the endothelial cells. Scientific research has proven that the depletion of levels of NO in circulation augments the adherence of monocytes and triggers the proliferation of smooth muscles, ultimately culminating in more damage to the vascular endothelium (19). Furthermore, according to the research conducted by Cai et al., possessing a higher BMI could imply a shorter duration of diabetes and better glycemic control. Individuals who are overweight and obese are at a greater risk of complications, hence necessitating a more thorough examination and care to deter the advancement of DR (20). However, more prospective studies with larger samples are needed to investigate the mechanisms involved.

The underlying causes of this negative correlation between hemoglobin levels and DR are still unknown. Despite the uncertainty surrounding the root cause of the negative correlation between hemoglobin levels and DR, this study’s findings can be elucidated through several mechanisms. Red blood cells in diabetic individuals exhibit reduced deformability and increased aggregation at the capillary level when compared to red blood cells in the general population (21–25), and are therefore more fragile and prone to rupture, thus reducing hemoglobin levels and possibly triggering anemia. Anemia may lead to ischemia, which is one of the key factors in causing DR (11). Under hypoxic conditions, anemia, a crucial sign of the body’s ability to transport oxygen (11), promotes the production of inflammatory mediators and angiogenic factors, such as vascular endothelial growth factor (VEGF) and erythropoietin (EPO) (26–28), which enhances retinal capillary permeability and promotes the formation of DR (13). The study revealed that VEGF can create dimer structures that interconnect with VEGFR-2 in the activation pathway of the receptor tyrosine kinase (29). This process leads to the induction of trans-receptor phosphorylation and the disruption of tight junctions, thereby improving vascular permeability (29–31). At the same time, VEGF can promote neovascularization by enhancing the hydrolysis of basement membrane proteins (32, 33) and levels of intercellular adhesion molecule 1 (ICAM-1) (34), inducing the development of more severe DR. Additionally, elevated blood VEGF levels sparked the creation of reactive oxygen species (ROS), which encourage apoptosis and activate endothelial cells, resulting in endothelial cell damage (28, 35). Intraocular EPO levels are significantly elevated in patients with ischemic retinopathy (e.g., PDR) (36–38), while *in vitro* studies have found that EPO can promote the proliferation of bovine retinal microvascular endothelial cells (BRECs) in a dose-dependent manner (38). The study confirmed that EPO acts as an ischemia-induced angiogenic factor that can also neovascularize (38).

This study has certain advantages, including the use of a survey database based on the US population, which improves the reliability and representativeness of the study. During the study, we adjusted for all covariates. However, the study still has some shortcomings. As this is a cross-sectional study, the causal relationship between hemoglobin levels and DR remains unclear. Furthermore, despite adjusting for several relevant potential covariates, the effect of other possible confounding factors could not be completely excluded. Moreover, the presence of diabetes and hypertension was confirmed by self-report, which may have been flawed.

## Conclusion

The present investigation reveals an inverse correlation between hemoglobin concentrations and DR, suggesting that decreased hemoglobin levels may exacerbate the development and progression of DR. Given these findings, medical professionals, healthcare providers, and policymakers must carefully contemplate the impact of low hemoglobin levels on DR when designing and implementing preventative and therapeutic measures for DR. On the one hand, monitoring of hemoglobin levels can be part of the routine follow-up of patients with diabetic complications, and on the other hand, interventions for low hemoglobin levels may also play a complementary role in reducing the risk of DR.

## Data availability statement

Publicly available datasets were analyzed in this study. This data can be found here: www.cdc.gov/nchs/nhanes/.

## Ethics statement

The studies involving human participants were reviewed and approved by the Research Ethics Review Board of the NCHS. The patients/participants provided their written informed consent to participate in this study. Written informed consent was obtained from the individual(s) for the publication of any potentially identifiable images or data included in this article.

## Author contributions

XL and MC designed the research. XL collected and analyzed the data, and drafted the manuscript. XL and MC revised the manuscript. All authors contributed to the article and approved the submitted version.
